# Resolution, access, and waiting time for specialties in different models of care

**DOI:** 10.11606/s1518-8787.2020054001627

**Published:** 2020-01-23

**Authors:** Natália Leite Rosa Mori, Jaime Olbrich, Regina Stella Spagnuolo, Carmen Maria Casquel Monti Juliani

**Affiliations:** I Universidade Estadual Paulista Faculdade de Medicina de Botucatu Programa de Pós-graduação em Enfermagem BotucatuSP Brasil Universidade Estadual Paulista (Unesp). Faculdade de Medicina de Botucatu. Programa de Pós-graduação em Enfermagem. Botucatu, SP, Brasil; II Universidade Estadual Paulista Faculdade de Medicina de Botucatu Departamento de Pediatria BotucatuSP Brasil Universidade Estadual Paulista (Unesp). Faculdade de Medicina de Botucatu. Departamento de Pediatria. Botucatu, SP, Brasil; III Universidade Estadual Paulista Faculdade de Medicina de Botucatu Departamento de Enfermagem BotucatuSP Brasil Universidade Estadual Paulista (Unesp). Faculdade de Medicina de Botucatu. Departamento de Enfermagem. Botucatu, SP, Brasil

**Keywords:** Health Services Needs and Demands, Basic Health Services, Primary Health Care, Referral and Consultation, Ambulatory Care Facilities, Hospitals, Specialties, Clinical Decision-Making

## Abstract

**OBJECTIVE:**

This study aimed to identify the treatment demands coming from primary health care units and, based on that, the demand for referrals to medical specialties in reference services. This study is justified by the scarcity of scientific literature on the subject.

**METHODS:**

This is a cross-sectional study using secondary data on the treatments and referrals made by the primary health care units, throughout 2014, in a municipality of the State of São Paulo, Brazil. The total population treated in 2014 was considered, resulting in 411,177 treatments.

**RESULTS:**

Out of all treatments performed, the percentage of referrals was of 4.42%, showing that 95,58% of the problems did not need to be referred to another service. A number of 8,897 referrals were made, to 6,850 users, who were mostly women (60.74%). The mean of referrals per patient was 1.3 (min. 1 and max. 8), and 1,604 patients (23.5%) were referred at least twice.

**CONCLUSIONS:**

Primary health care services have been responsible for a large number of treatments, whereas the demand for referrals has decreased, suggesting that such services have established themselves as a gateway to the health system and achieved the expected solvability, although the waiting time for some specialties is very long.

## INTRODUCTION

The global context of primary health care is promising, showing successful results in some of the implemented models. However, for this success to be achieved, an efficient planning is required, as well as the realization of public health programs and the cooperation of the population^1^.

Among its advantages, primary care has shown efficiency of health services, low expenses, reduced needs for urgency and emergency care, and good user satisfaction. It is a highly effective way to address the main causes of diseases and their risk factors, and to handle the challenges that may threaten the patients’ health in the future. Nonetheless, presenting such results, primary care should be able to meet the health needs of the population and solve its problems as demanded^2^.

In Brazil, the health services are predominantly built based on the traditional model of care, which contributes to the persistence of fragmented actions, centered in the complaints and in the biological aspects of the patient. This poses challenges for the consolidation of the health care model currently proposed^5 , 6^.

The concept of Primary Health Care has been repeatedly reinterpreted and redefined, generating confusion around the term^4^. The modern meanings of the terms “Basic Care” and “Primary Health Care” are equivalent, and all establishments providing Basic Care services and actions within the Brazilian Unified Health System (SUS) are called Basic Health Units (BHU)^7^.

Despite the similar name, some differences are observed depending on the health model adopted: the Family Health Units (FHU) have a determined coverage area, assisting families registered within it, and rely on teams of professionals defined by the Ministry of Health; whereas traditional units, the so-called Basic Health Units (BHU), have different distributions and count on medical professionals (clinicians, pediatricians and gynecologists/obstetricians), nurses, dentists, and nursing assistants, in addition to the possibility of support from some specialists, in areas such as Ophthalmology and Dermatology^8^. Some municipalities also have services with specific characteristics, such as the “Health School Centers” (HSC), which are associated with Health Sciences colleges and establish a similar model to that of the basic units, but with greater autonomy.

In the Brazilian scenario, the Ministry of Health considered that primary health care units should solve at least 80% of the population’s health problems. That is, they should not send more than 20% of the demand to specialized services^9^.

Among the strategies to achieve a better health care management is the recognition of potential and effective provisions of services^10^. Thus, the solvability capacity of a unit or model can be evaluated by various approaches, such as service demand, coverage, and access by the population. However, what really shows whether the patient’s health problem was solved or not is their referral^11 , 12^.

Despite the important role played by referral systems in many health care systems, surprisingly few of them have been evaluated, resulting in a limited evidence base to support decisions^13^. There is little literature on treatments and referrals in primary care that shows and describes the reality of a Brazilian municipality or even of the Brazilian population, especially due to the predominance of qualitative studies^6 , 11 , 14^.

Studies that compare the vacancies and waiting times for the access to specialized health services are scarce, especially those associating different models of primary care. Therefore, this research sought a quantitative approach to the solvability of primary health care services in a municipality of the countryside of SP.

## OBJECTIVE

We sought to analyze the following aspects, in a city of the countryside of São Paulo (Brazil): the solvability of primary health care and of different models of care; the referrals generated; the waiting times for medical care, referral, and scheduling of the consultation in the specialty; and the related demographic aspects.

## METHOD

This was a population-based cross-sectional study, using retrospective secondary data, collected from primary health care services in a municipality of approximately 138,000 inhabitants, located in the State of São Paulo (Brazil). The total population treated in 2014 was considered.

Data were collected between January and August 2016 at the Information Technology Center of the Municipal Secretariat of Health. A password was created to access the System reports and collect the information necessary for the development of the survey. We gathered the information on the services rendered from January 1 to December 31, 2014.

All Primary Health Care Units of the Municipality in operation during this period were included, totaling: 11 Family Health Units, 6 Basic Health Units, and 2 Health School Centers.

We considered treatments performed by any of the members of the health team, except those done by dentists. For the purpose of evaluating service solvability, we took into consideration the consultations by medical, nursing, or psychology professionals, since these are the professionals who can request referrals to medical specialties.

In the municipality, these professional categories make referrals using an instrument containing the patient’s personal data, the reason for the referral request, the specialty to which the patient is being referred, among other information. Along with this instrument, the professionals register the requests in the Information System. Both the record and the instrument are received by the Municipal Secretariat of Health, which verifies the data, analyzes the case, and inserts it in the waiting line for a consultation with an expert, seeking to prioritize more severe cases.

The patients may be referred to different specialties, but only one time to each; they can only be referred to the same specialty again after scheduling or canceling the consultation (due to non-attendance, for example).

The offer of vacancies for medical specialties is not predetermined, therefore, we used information from the Municipal Secretariat of Health, which included all the offers and referrals during the studied period. Other data were extracted from the management reports of the Viver^®^ Information System, kept by the same Secretariat, which stores the information on all treatments carried out in primary health care services of the municipality, and regulates the access to reference services.

The specialties that have not been addressed, such as Psychiatry, for example, are those with “free demand” access, in which the demand is met according to the model of differentiated flow, being impossible to track them by the Information System used. Based on the data collection period, we could identify the waiting time for the treatment with specialists and to follow the outcomes up to 541 days after the request for a referral, when the period of data collection was closed. All referrals made after this period were classified as having a waiting time “greater than 541 days”.

The statistical analysis was carried out in the SAS^®^ software for Windows, using descriptive analysis, cross tables, and R for difference tests of Chi-square type proportions.

This project was submitted to the authorization of the involved institutions and was approved by the local Research Ethics Committee, under the CAAE No. 43031615.2.0000.5411 on 04/06/2015.

## RESULTS

In 2014, 411,177 treatments were performed, of which 268,046 (65.19%) served women and 143,131 (34.81%) served men, the mean age of the patients was 39.46 years – ranging from 0 to 105 – in a total of 66,833 patients treated. The mean number of treatments per person was 6.15; with a minimum of 1 and maximum of 269 appointments over the period.

Physicians, nurses or psychologists were responsible for 201,220 consultations, 48.93% of the total number of appointments. 8,897 referrals were made, for an amount of 6,850 users, mostly comprising female patients (60.74%). The mean number of referrals per patient among those who needed referrals was 1.3, and 1,604 people were referred at least twice (23.5%), with a variation from 1 to 8 times. The mean age of the referred patients was 48.21 years, with a minimum of 1 and maximum of 99 years of age.

The mean percentage of referrals considering the total of treatments performed was 4.42%, showing that 95,58% of the problems did not need to be referred to another service. This may indicate resolutions, but that cannot be tacitly affirmed, since there was no follow-up of the cases. When analyzed separately, the different models of care presented significant differences regarding the proportion of referrals, with solvability percentages that ranged from 92.5% to 98.24% in the FHU, from 93.76% to 96.79% in the BHU, and from 92.58% to 96.18% in the Health School Center (HSC) model.

**Graph 1:**
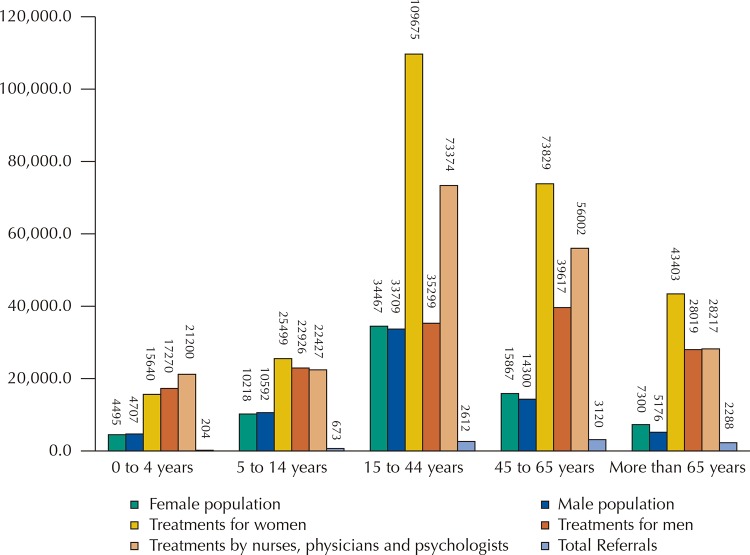
Population, number of treatments and referrals, by age group and sex, in a municipality of São Paulo, 2014.

In the municipality, the population distribution by age group^15^and sex showed the relationship between the total patient population and the number of treatments and referrals of such population ( Graph 1 ).

The waiting time after the consultation that generated the referral, until its scheduling in the reference service also presented significant differences (p < 0.0001) among the health care models, as shown in Table 1 . No difference was observed among the referrals that took more than 540 days to be scheduled. Similarly, there was no significant disparity between the BHU and the FHU for referrals with waiting time between 361 and 540 days. The other time tracks, however, presented *p* < 0.0001 from one another.

**Table 1:** Waiting time between the referral from primary health care to specialty care and the scheduling of a consultation with the specialist, in a municipality of the State of São Paulo, 2014.

Waiting time (days)	HSC	%	BHU	%	FHU	%	Total	p-value
0 to 60	1832a	73.4	2192b	61.5	1692c	59.6	5716	<0.0001
61 to 120	407a	16.3	814b	22.8	719c	25.3	1940	<0.0001
121 to 180	116a	4.6	260b	7.3	209c	7.4	585	<0.0001
181 to 360	97a	3.9	233b	6.5	169c	6.0	499	<0.0001
361 to 540	20a	0.8	42b	1.2	30b	1.1	92	0.0026
541 or more	23	0.9	23	0.6	19	0.7	65	0.6913
**Total**	**2495**	**100.0**	**3564**	**100.0**	**2838**	**100.0**	**8897**	

Observation: The same letter does not differ by the proportions difference test.

The number of referrals to distinct specialties were different according to the care model of the primary health service that generated the referral, as shown in Table 2 . Dermatology was the most requested specialty by all care models, whereas Orthopedics and Traumatology were more requested by traditional Basic Units than in the two other models. The Allergology specialty was the less requested one, regardless of the care model.

**Table 2:** Vacancies offered, and referrals made by primary health care services, in a municipality of São Paulo, by specialty and health care model, in 2014.

Specialty	Referrals				Vacancies/ year	% Coverage

	HSC	BHU	FHU	Total		
Allergology	11	15	12	38	2	5.26
Cardiology	274	220	247	741	197	26.59
Dermatology	527	993	503	2023	738	36.48
Endocrinology and Metabolism	24	71	65	160	3	1.88
Gastric Surgery	83	56	57	196	73	37.24
Gastroenterology	119	204	130	453	107	23.62
Gynecology and Obstetrics	59	227	178	464	254	54.74
Hematology	90	10	8	108	29	26.85
Nephrology	20	37	53	110	48	43.64
Neurosurgery	71	53	41	165	67	40.61
Neurology	220	311	239	770	245	31. 2
Ophthalmology	109	282	345	736	384	52.17
Orthopedics and Traumatology	203	405	293	901	183	20.31
Otorhinolaryngology	321	241	205	767	47	6.13
Plastic Surgery	34	69	55	158	8	5,06
Pneumology	4	51	42	97	27	27,84
Rheumatology	50	53	68	171	15	8,77
Urology	110	205	220	535	217	40,56
Vascular Surgery	166	61	77	304	39	12,83
**Total**	**2495**	**3564**	**2838**	**8897**	**2683**	**30.15**

**Graph 2:**
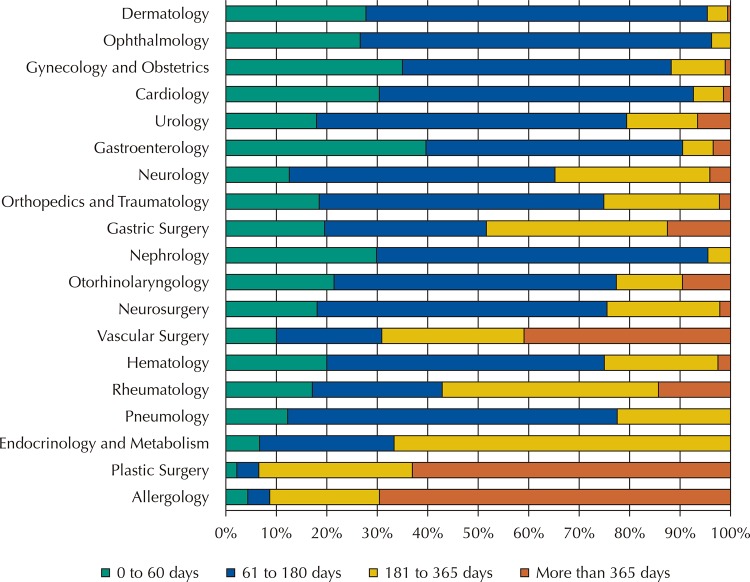
Referrals made in the primary health care according to specialty and waiting time, in a municipality of São Paulo, 2014.

As for the amount of time needed for the scheduling of consultations referred by the primary care, the mean was 122.85 days (mode and median: 85), ranging from 1 to 918 days. The time taken to access the consultation in the different specialties was heterogenous, as demonstrated in Graph 2 . Some specialties answered more than 90% of demands within 180 days, such as Dermatology, Ophthalmology, Cardiology, Gastroenterology, and Nephrology, whereas for Allergology, Immunology, and Plastic Surgery, more than 50% of the demand had a waiting time greater than 365 days.

## DISCUSSION

The treatment and referral of female patients were found prevalent in this study, corroborating with data from other researches^9 , 11 , 16^. The low rate of male demand for treatments in health services may be associated with the form of organization of these services – their hours of operation match the workhours –, and to sociocultural barriers associating the demand for care to an idea of greater vulnerability^9 , 10^.

The results of this study reaffirm the profile of the user population of the primary health care services established by other researches^9 , 11 , 17^, and define the referral rates of all studied units as appropriate to the recommended rate of 20%. Such results reinforce the importance of the primary health strategy and of the Family Health Care model, which showed the lowest average rate of referrals among the three methods analyzed.

The rate of referrals made by primary health care services (4.42%) was similar to those in available data, which were between 2.7% and 8.6% in national studies^8 , 10^and equaled 8.54% in an international research^17^, thus reinforcing the understanding that the primary care, when organized and effective, can solve from 87.5% to 91% of its demand^18^.

Despite the close values, there was a significant difference among the referrals made by primary health services according to the care models adopted. This information confirms a previous study which also noted a significant difference between the referrals made in FHU and in traditional units^9^. It is difficult to come to general conclusions regarding the applicability of these findings, given the marked differences of health systems from one place to another^19^.

It is worth noting that referrals from the primary care to specialized care services are largely determined by the prior experience in the management of certain diseases; hence, the referred demand also depends on the specialty or expertise of the physician working in primary care^17^. Among the main organizational interventions that may improve referral rates and referral appropriateness are obtaining a second in-house assessment of referrals and having appointment slots dedicated to secondary level appointments in all primary care practices ^19^.

Professionals who perform treatments in primary health care must be accurate and capable to diagnose specific problems that require specialized treatment, avoiding late referral and problem worsening. Professionals must be able to diagnose and manage health problems from their beginning in order to avoid the patient’s dependence on special care, and provide more appropriate care to chronic health conditions^20^.

For these data to represent what they suggest, however, the access to basic service must be ensured to all because, if the treatment coverage is low, the high solvability is only apparent. We must consider that it was only possible to quantify the number of treatments and referrals of patients who had access to the health service. Since the estimated population of the studied municipality was of 138,019 inhabitants in 2014^15^, and 66,833 patients were treated in the primary care services, then 48.42%, less than half of the population, had access to the System.

Analyzing Graph 1 , we could understand that primary care has strictly fulfilled its role. However, we must be cautious to explore the circumstances of the consultations performed and pay attention to the pattern formed by the patients. Considering that a single patient attended the health services 269 times, we can assume that the demand for primary care is often focused on a small portion of the population.

Admitting that women in childbearing age, which make up the largest portion of treatments detected, are more frequently treated for obstetrical and gynecological needs^18^, we must assume that the professionals and health service structures are prepared to meet this demand. They should also be prepared to promote preventive and educational actions targeted at the male population, since its demand for health care services diminishes in this age group, increasing again at older ages, when the health problems are already aggravated.

In this context, the fact that the “Health School Centers” were the services that most referred patients to medical specialties and, at the same time, had the lowest waiting time for scheduling such consultations, may suggest that the proximity between Health Unit and University motivates referrals to specialties and influences the regulation by the early scheduling of such referrals. The primary care/specialist interface can be an organizational key feature for many health care systems, whereas the presence of experts working at the primary health care level may help in the detection of specific diagnostic suspicions and accelerate the referrals^13 , 21^.

A study conducted in Ethiopia showed that the users of health services had a routine of going to hospitals with no reference and without previously seeking other sources of care, such as primary healthcare units or health centers, despite the remarkable expansion of such services in the country^22^. In Canada^23^, in turn, it was shown that people with higher educational levels tended to skip steps more often, rarely starting from primary health care to reach specialized care services.

The long waiting time for consultations with specialists can lead to the aggravation of diseases, to emergency hospitalizations, and to an overwhelm of the public health system^24 , 25^.

The data presented reveals a nonconformity between the offer of vacancies for appointments with specialists and the demand of patients for such services. Together with the circumstantial lack of specialized care, we must resume our argument on the access to health services – they are receiving less than half of the population, that is, the Brazilian Unified Health System (SUS) health services have not provided enough coverage for the entire population.

In this study, Dermatology was found to be the specialty with the highest number of referrals and the greatest amount of vacancies. However, given the proportions between both, it had a low percentage of coverage (referrals/vacancies). Comparing health care models, Ophthalmology was requested the most at Family Health Units, which can be explained by the presence of expert professionals in some other services and by the role of FHU in the expansion of the access to health services, enabling the emergence of a previously hidden demand.

The expansion of primary care has been implemented to ensure access to health services. However, it is limited to gateway services, not being extended in an equivalent proportion to services of higher technological density, which could absorb the referred demand. To meet the health needs of the population, it is necessary to define the expertise of professionals in clinical practices and their functions, so that such a complex health system works^26^.

It is worth considering that the delay in treatments could be a regulatory barrier within the health system, with the long waiting time becoming a device to restrict the access in a universal system. It is possible that, after waiting for long, the patient searches other alternatives to solve their problem^5 , 24^.

Although strategies to diminish the waiting time may seem simple, there is no way to draw feasible solutions without knowing the demands of the target population and the time it takes to obtain treatment. The collection and reporting of such data are not enough to organize the demand and reduce the waiting time but are necessary to outline goals and make strategic decisions for planning and managing the Health System^21 , 27^.

To improve the work process organization and achieve referral equity, it is important that protocols and therapeutic guidelines are defined for the prioritization of cases^14 , 21 , 25^. Planning actions with an effective use of health management and regulation tools is vital for the control of offer and demand in health services.

The local character of this research can be considered a limitation, but we believe that these results can be comparatively extended to other locations, if the peculiarities of each health service are taken into account. They may also be used to understand the demands in the different health care models and to instigate questions, from which further researches could arise.

## CONCLUSION

The profile of the demands met and referred in the different health care models has the potential to contribute with an estimate of resources needed to meet them and with the analysis and organization of health services.

These results respond to our proposed objectives and reinforce the idea that the expansion of primary health care services has not been accompanied by the expansion and restructuring of other levels of care. Even if the patients are referred to experts, the response to their problems can only be assured at the consultations with these professionals , which may not be effective if the waiting time is too long.

It is not possible to affirm that the low rate of referrals implies a high demand solvability by the primary health services, since the cross-sectional cut of this study did not allow patient monitoring. This means that a more in-depth debate and the development of further research on the topic would be necessary.

We must reflect on the practice of specialized services and consider regulatory protocols to aid the effective communication among services, which enable networking, in order to provide comprehensive health care services that can respond equally to the health needs of the population.
